# The effects of community-based interventions on the uptake of selected maternal and child health services: experiences of the IMCHA project in Iringa Tanzania, 2015‐2020

**DOI:** 10.1186/s12884-023-05638-x

**Published:** 2023-05-08

**Authors:** Stephen Oswald Maluka, Chakupewa Joseph Mpambije, Peter Clever Kamuzora, Sian Fitzgerald

**Affiliations:** 1 Institute of Development Studies, University of Dar Es Salaam, P.O. Box 35169, Dar Es Salaam, Tanzania; 2 Dar Es Salaam University College of Education (DUCE), P. O Box 2329, Dar Es Salaam, Tanzania; 3 Department of Development Studies, History and Political Sciences, Mkwawa University College of Education (MUCE), P.O. Box 2513, Iringa, Tanzania; 4 Healthbridge Foundation, Ottawa, Canada

**Keywords:** Community-based interventions, Participatory women groups, Maternal and childhealth

## Abstract

**Background:**

Maternal and child health (MCH) improvement has been prioritised in resource-constrained countries. This is due to the desire to meet the global sustainable development goals of achieving a maternal mortality rate of 70 per 100000 live births by 2030. The uptake of key maternal and child health services is crucial for reducing maternal and child health mortalities. Community-Based Interventions (CBIs) have been regarded as among the important strategies to improve maternal and child health service uptake. However, a paucity of studies examines the impacts of CBIs and related strategies on maternal and child health. This paper unveils the contribution of CBIs toward improving MCH in Tanzania.

**Methods:**

Convergent mixed method design was employed in this study. Questionnaires were used to examine the trajectory and trend of the selected MCH indicators using the baseline and end-line data for the implemented CBI interventions. Data was also collected through in-depth interviews and focus group discussions, mainly with implementers of the interventions from the community and the implementation research team. The collected quantitative data was analysed using IBM SPSS, while qualitative data was analysed thematically.

**Results:**

Antenatal care visits increased by 24% in Kilolo and 18% in Mufindi districts, and postnatal care increased by 14% in Kilolo and 31% in Mufindi districts. Male involvement increased by 5% in Kilolo and 13% in Mufindi districts. The uptake of modern family planning methods increased by 31% and 24% in Kilolo and Mufindi districts, respectively. Furthermore, the study demonstrated improved awareness and knowledge on matters pertaining to MCH services, attitude change amongst healthcare providers, and increased empowerment of women group members.

**Conclusion:**

Community-Based Interventions through participatory women groups are vital for increasing the uptake of MCH services. However, the success of CBIs depends on the wide array of contextual settings, including the commitment of implementers of the interventions. Thus, CBIs should be strategically designed to enlist the support of the communities and implementers of the interventions.

**Supplementary Information:**

The online version contains supplementary material available at 10.1186/s12884-023-05638-x.

## Introduction

Efforts to reduce maternal and child mortality have remained a top priority at the national and international levels. As such, signs of maternal and child mortality reduction have been witnessed globally. For instance, between 2000 and 2017, the maternal mortality rate was estimated to have been reduced by 38% [1]. Despite the reduction in maternal mortality, the World Health Organization (WHO) estimates that globally, in 2020, about 800 pregnant women died every day due to preventable complications related to pregnancy and childbirth [2]. About 95% of these deaths occurred in Low and Middle-income Countries (LMICs), with Sub-Saharan Africa (SSA) accounting for almost 70% of all maternal deaths. Also, in 2020, SSA countries recorded the highest rate of neonatal and under-5 mortality in the world, with 27 newborn deaths per 1000 live births, accounting for 43% of global newborn deaths [3, 4]. Evidence still portrays challenges for realising the Sustainable Developments Goal 3, targets 3.1 and 3.2.2 aiming at reducing maternal deaths to less than 70 per 100,000 live births and neonatal deaths to 12 per 1000 live births by 2030, respectively [5].

Evidence suggests that improving adherence to maternal and neonatal health continuum of care protocols, including antenatal care (ANC) and postnatal care (PNC) services, can reduce the burden of maternal and neonatal mortality [6–8]. ANC offers a lifesaving opportunity by timely detecting danger signs and reducing complications while addressing health care inequalities in remote and marginalised settings [9–12]. Similarly, the timely and appropriate undertaking of PNC improves child survival, reduces morbidity, and improves the quality of life [13]. It also encourages proper hygiene and nutrition, exclusive breastfeeding, breast care, birth spacing, safe sex, and promotes a healthy lifestyle [6].

While attendance of ANC is highly promoted, the uptake of these services in LMICs is still low. At the global level, for instance, 71% of pregnant women receive ANC services, and 95% of them are from developed countries, while only 69% are from SSA [14, 15]. Data shows further that in LMICs, early initiation of ANC in 2013 stood at 24% compared to 81.9% in developed countries [16]. The status of ANC uptake in Tanzania is also not impressive, as only 24% of pregnant women attend ANC early enough, while 51% attend four or more times [17]. Similarly, the uptake of PNC in Tanzania is reasonably low, ranging from 25% in 2010 to 34% in 2015. This was far below the national target, which required 80% of mothers and newborns to receive early PNC check-ups by the year 2020 [18]. The evidence remains relatively low for family planning, which stood at 20%, 27%, and 32% in 2004, 2010, and 2015, respectively [17, 18].

Community-based interventions (CBIs) such as participatory women groups and home visits by trained community health workers (CHW) have shown effectiveness in reducing the burden of maternal and child mortality and morbidity in LMICs [19–22]. A body of evidence asserts that Women’s Groups (WG) interventions can improve the uptake of ANC and PNC services [23, 24]. Most CBIs have employed socio-ecological approaches to reach pregnant women through (a) group-based discussions and/or household visits [25] and (b) fostering supportive social norms by engaging household influencers, particularly male partners who often make decisions on behalf of pregnant women [26, 27]. Most studies on WG interventions have been generated in South Asia, with very few studies emerging from the SSA [21]. For example, a few studies in SSA have been conducted in Malawi [28], Zambia [29], Uganda [30] and Kenya [25]. In addition, most studies assessing the impacts of the implemented WG interventions used quantitative data, with only a few studies employing mixed methods. Against this background, this study examined the effects of CBIs through WGs implemented in Iringa, Tanzania, from 2015 to 2020 under the Innovating for Maternal and Child Health (IMCHA) programme. This paper adds to the body of knowledge that articulates the importance of utilising WGs to improve MCH in resource-constrained settings.

## Methodology

### Study design and setting

This study employed a convergent mixed method design to examine the effects of WG interventions towards improving MCH. Mixed methods facilitated the triangulation of findings to gain a complete picture of the explored issues [31]. In addition, the design helped to provide diverse information and detailed views of the participants [32, 33]. This study was conducted in Kilolo and Mufindi districts in Iringa Region, where a large project was being implemented under the Innovating for Maternal and Child Health in Africa (IMCHA) programme (2015**-**2020) was implemented. The two districts were selected because they exhibited an unacceptably low uptake of maternal and child health services, including ANC, PNC and FP services. For instance, in Kilolo and Mufindi districts, attendance of ANC within twelve weeks (first trimester) was 16.8% and 27%, respectively; and pregnant women who completed four ANC visits and above in 2018 stood at 27.1% in Kilolo and 23% in Mufindi [34, 35]. Apart from exhibiting low MCH uptake, the two districts also experienced weak and inadequate health systems. For example, the shortage of health workers was 39% for Kilolo District and 38% for Mufindi District [36–38]. In addition, the districts are predominantly rural, which may have hindered their access to maternal and child health services, which calls for the need to implement the CBI interventions [37]. Table 1 shows the key characteristics of the study settings.

**Table 1:** Key characteristics of the study settings

	**Kilolo district**	**Mufindi district**
Population	218,138	246,090
Population Growth Rate	1.4%	1.5%
Hospitals	1	1
Health Centers	2	8
Dispensaries	56	45
Divisions	3	5
Wards	24	27
Villages	106	121
Health workers available	61%	62%
ANC within 1^st^ trimester as of 2016	16.8%	27%
Completion of four or more ANC visits as of 2016	27%	23%

Source: CCHP Mufindi and CCHP, 2018

According to the Comprehensive Council Health Plan, 2018, Kilolo District was supposed to have 1247 health workers, but only 756 were available, while in Mufindi district, the ideal number of health workers was expected to be 1326, but only 822 were available. This indicates that of the two districts, none had the required number of health workers.

### IMCHA project: the intervention

The IMCHA project was implemented in Kilolo and Mufindi districts from 2015 to 2020. The overall project aim was to develop, adopt and implement CBIs to improve maternal, newborn and child health outcomes to inform policy and scale-up in Tanzania. Participatory Action Research (PAR) through women's groups intervention in collaboration with the Implementation Research Team (IRT) worked with community members to address MCH challenges. The PAR was implemented through a series of meetings with implementers of the interventions, namely Women Groups (WGs), Male Champions (MCs), Women Group Supervisors (WGS), village leaders, community gatekeepers and health care providers. The PAR was facilitated by the IRT from the University of Dar es Salaam and health managers from Iringa Region in collaboration with the HealthBridge Foundation of Canada. Details of PAR phases and the series of meetings through which the IMCHA project was implemented are described elsewhere [36, 38]. Overall, the intervention was implemented in four phases as follows: Phase I covered the identification of MCH problems in the respective villages and prioritised the most important 3-5 MCH problems affecting the community. Phase II involved developing strategies to address the prioritised MCH problems. The proposed strategies were presented to the community meetings by WGs involving different stakeholders, namely government and community leaders, religious leaders, health care providers, and health facility governing committees. In phase III, WGs implemented strategies to improve MCH services. The dominant strategies included community sensitisation meetings, household visitations of pregnant women, and engagement of male champions and community gatekeepers through religious and village elders. Healthcare providers (HCPs) were also consulted. In Phase IV, the implementers of interventions evaluated the effectiveness and sustainability of the implemented strategies. The project was anchored on the perspective that integrating the participation of key community actors would influence the uptake of crucial maternal and child health services.

### Selection of study site and participants

The study was conducted in health facilities in Kilolo and Mufindi districts where WG interventions were implemented. Five wards were purposively selected in each district as they had implemented the project. In each ward, two villages were selected for the study as they also implemented the WG interventions. From the selected villages, 13 health facilities from 10 wards were included. From the selected health facilities, baseline and end-line data were collected to determine the trajectory and trend of the MCH before and after the interventions. Participants in this study were purposively sampled from those who directly participated during the implementation of the interventions. As indicated in Table 2, 86 participants were involved in the study.

**Table 2:** Categories and number of respondents

No	**Category**	**Number of respondents**
1	Women Group members	30
2	Male champions	10
3	Women group supervisors	10
4	Village management team members	10
5	Health care workers	8
6	Community gatekeepers	6
7	Council Health Management Team members	8
8	Implementation research team members	4
	**Total**	**86**

### Data collection techniques

Data was collected through closed-ended questionnaires, in-depth interviews (IDIs), Focus Groups Discussions (FGDs) with participants, and document reviews. The questionnaires were administered to the in charge of each health facility in the respective wards where interventions were implemented. The questionnaires were structured to capture some MCH outcomes like ANC, FP, PNC, and male involvement during the five years of implementing the project. These data revealed the trajectory and trends of the selected MCH indicators. Researchers collected the baseline data using the questionnaire (Additional file 1) to map the monthly trend of some selected MCH outcomes in 2016. This was followed by the end-line data collected in 2019 after the implementation of the interventions to determine whether there were any trends and trajectories of changes experienced after the implementation. Finally, in-depth interviews (IDIs) sought to collect detailed information regarding the efficacy of the implemented interventions in improving some MCH services.

Interview guides were developed after reviewing the IMCHA technical reports and the grant proposal that sought to address the contribution of WG interventions in improving the uptake of key MCH services. The research team comprised experts in public health, community participation and the application of quantitative and qualitative methodologies. The researchers' SM, CJM and PK conducted IDIs in Kiswahili, lasting between 45 min and 1 h. The interviews were digitally recorded with permission from the participants. After each research phase, which took two to three days, the research team held briefing sessions on important ideas and themes from participants, including key summary points and any emerging information. The collected information revolved around the impact of the implemented interventions in the study areas. The primary impact revolved around the uptake of modern contraceptive methods, ANC and PNC visits. Secondary impact revolved around the changes experienced after the intervention's implementation, which could be at the community level among the intervention's implementers and health care providers. The interviews were conducted at the participants' homesteads or workplaces from June to September 2019.

In addition, six FGDs were conducted, three in each district, with an average of 8–10 participants totalling 48 FGD participants. Out of the five wards in each district where WGs interventions were implemented, the FGDs were conducted in purposively selected three wards, making six wards out of 10 in the two districts. As FGDs sought to ascertain the efficacy of the implemented interventions, they involved members from WGs and WGS who were actively engaged during the implementation of WG interventions. The FGDs enabled the participants to critically discuss their experiences during the implementation of the interventions as well as the primary and secondary impacts of the implemented interventions.

A review of documents was also conducted. Because the study was not leaning on a systematic review process, scoping was opted for. Thus, a purposive selection of relevant literature and information was preferred. Reviewed documents included district annual health plans for 2017 and 2018, which provided information on the status of MCH and overall health service provision in the two districts and the IMCHA documents, which included project strategic and action plans, formative research reports, workshop reports, and project progress reports. These reports provided a good opportunity to gain a detailed understanding of the impacts brought about by the implemented interventions. The review also captured published and unpublished documents related to the implementation of CBIs in LMICs. Two authors (SM) and (CJM) were responsible for the literature search, after which they shared the documents accessed with all authors for use during their inputs in manuscript development.

### Data analysis

Qualitative data was analysed using thematic analysis [39]. The audio recordings from IDIs and FGDs were transcribed verbatim by trained transcribers and then translated from Kiswahili into English. The core research team members reviewed the transcripts and made notes for each transcript. First, the Principal Investigator (SM) developed a code manual based on the study's objectives. The coding framework was further discussed and refined by the research team members. Next, PK, CJM and SM manually coded the transcripts. Additional themes and sub-themes which emerged during the coding process were added along the way. Responses were then compared across different types of participants. Finally, data was summarised and synthesised, keeping the key expressions of respondents as illustrative cases. Quantitative data analysis was performed using the IBM SPSS statistical software 20.0 to generate descriptive statistics.

### Ethical considerations

This study got approval from the Ethical Committee of the National Institute for Medical Research of Tanzania (NIMR) withcertificateNo: NIMR/HQ/R.8a/Vol.IX/2119. The Regional Administrative Officer in the study region also approved the study. Moreover, verbal informed consent was obtained from all participants. Verbal consent was preferred to written consent because it was assumed that asking respondents and participants to sign consent forms would create fear and thus discourage them from participating in the research. Similarly, data was only accessible to the team members; and no individual identification was attached to the findings. Lastly, all quotes used to illustrate participants’ views had no personal identities.

## Results

This section presents the significant findings of the study. The results revolve around two major themes: primary improvement of selected major MCH services and secondary improvement of MCH services. The primary theme had five sub-themes: attendance of ANC within the first trimester, four or more ANC visits, male involvement in MCH services, uptake of PNC and use of modern contraceptives. The secondary theme had three sub-themes, including awareness and knowledge of matters pertaining to MCH, attitudes among healthcare workers, and empowerment of women group members.

### Demographic features of the study participants

As indicated in Table 3, females dominated the study, accounting for 47(54%), while males were 39 (46%). Women groups comprised 30 (34.8%) females with 10 (11.6%) male champions, while community gatekeepers were 6 (6.9%). Other study participants included 10 (11.6%) women group supervisors, 10 (11.6%) village management team and 10 (11.6%) health care workers. Concerning the age of the participants, the majority were aged 30–39 years, followed by the age group of 40–49, a few were 50–59 years, and no participant was 60 years or above. The analysis of the education of the participants revealed that the majority of WGs, 22 (73%), were primary school leavers, 2(7%) had no formal education, and 6(20%) had secondary education. Again, 6 (60%) of male champions had attained primary school education, 3(30%) secondary education, and 1 (10%) had a certificate. Also, 5 (50%) of WGS had acquired primary education, and 4 (40%) had a secondary level of education. All healthcare workers had post-secondary education with certificate and diploma levels, especially in health-related fields.

**Table 3:** Demographic features of the study participants

Category	Sex		Age range (years)				
	Male	Female	20–29	30–39	40–49	50–59	60 +
**Participants from the Community**							
WGs	N/A	30	08	10	09	03	-
MCs	10	N/A	01	03	04	02	-
CG	04	02	-	-	01	03	02
WGS	04	06	02	03	05	02	-
VMT	07	03	01	05	02	02	-
HCWs	05	03	-	04	03	01	-
Total	30	44	12	25	24	13	02
**Project Technical Personnel**							
CHMT	05	03	-	02	04	02	-
IRT	04	-	-	00	02	01	01
Total	09	03	-	02	06	03	01

Source: Field data, (2019)

Key: *WGs* Women Groups, *MCs* Male Champions, *CGs* Community Gatekeepers, *WGS* Women Group Supervisor, *VMT* Village Management Team, *HCWs* Health Care Workers, *CHMT* Council Health Management Team, *IRT* Implementation Research Team

### Primary improvement of maternal and child health services

The implementation of CBIs through WGs targeted the improvement of MCH services like ANC, PNC, uptake of family planning and male involvement primarily. The following section describes how the implemented interventions improved that continuum of care.

#### Pregnant women who sought ANC services within the first trimester

Participants articulated an apparent increase in the number of pregnant women who started ANC visits during the first trimester, at the 12^th^ week of pregnancy. Participants asserted that before the implementation of the interventions, few pregnant women used to attend ANC on time. To testify to the observed changes, one healthcare provider stated thus:

*The WGs played an extraordinary role in increasing the number of pregnant women who come for ANC services. In the last three years, more women have sought services at this facility. You know, women in the village do not think it is important to go to a health facility, especially in the 1*^*st*^* trimester, especially if one is feeling well. We have been involved in this project and recorded significant changes in women's attitudes as more and more attend ANC voluntarily and without expecting a looming health challenge* (IDI with HCP, in KDC).

Village leaders reported having witnessed similar changes in their respective villages; as one village leader rightly put it:

*When I reported to this village during my first appointment, I received many complaints regarding women who attended ANC very late. These days, however, things have changed for the better. I can now stay even two months without hearing such complaints* (IDI with VMT, in MDC).

During FGDs, participants echoed the same views regarding the changes they had witnessed:

*Since we started sensitising communities, much has been witnessed regarding the early attendance of ANC. Our approach of identifying pregnant women who have not attended ANC on time and accompanying them to the facility has changed everything. So, we have reasons to celebrate* (FGD with WG, MDC).

**Fig. 1:**
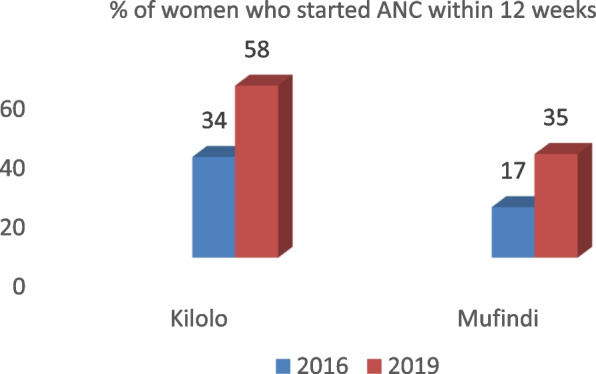
Increased ANC uptake within 1^st^ trimester

The revelations from the participants above were in congruence with evidence from health facilities. As indicated in Fig. 1, the data collected from facility registers showed that the rate of pregnant women who started ANC within 12 weeks increased by 24% and 18% in Kilolo and Mufindi districts respectively.

#### Pregnant women who completed four or more ANC visits

**Fig. 2:**
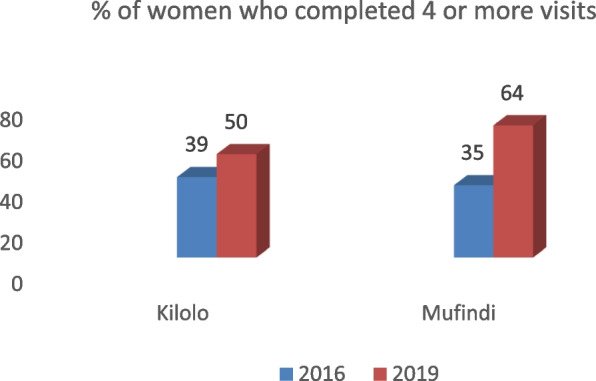
Pregnant women who completed four or more ANC Visits

Findings from the study revealed an increase in the number of pregnant women who completed four ANC visits. It was revealed that as many pregnant women started ANC on time, they continued to attend subsequent ANC clinics. Additionally, it was reported that sensitisation meetings through folk media underscored the importance of completing four ANC visits and the risks associated with failing to complete all the visits. The data extracted from the facility registers indicated that the percentage of pregnant women who completed 4 or more ANC visits increased by 11% and 29% in Kilolo and Mufindi districts, respectively, as Fig. 2 illustrates.

Evidence from the health facilities further revealed that of all ten health facilities, seven showed a positive increase of pregnant women completing four or more ANC. In contrast, two health facilities did not record any changes in the number of women who completed four ANC visits. In particular, the facilities that recorded the highest increase in ANC visits were Itungi in Kilolo District, with a 37% increase, and Kibengu in Mufindi District, with an increase of 35%. In congruence, during interviews, participants reaffirmed the reported changes as stated by one of the participants;

*Indeed, at my facility, I have visibly witnessed an increase in the number of women completing all four visits and above. There is a clear difference between the time before the implementation of the intervention and after the intervention. The sensitisation of the communities through public meetings, some of which I happened to attend, has contributed to the increased number of pregnant women who now complete four visits at this facility* (IDI with health worker in MDC).

In support of this view, WG members reiterated during FGD sessions that the WGs approach to the implementation of interventions revitalised pregnant women to complete four ANC visits. This was elaborated by one WG member thus:

*Of course, there was a tendency of slumbering among pregnant women when it came to the completion of all four visits. But our efforts have enabled them to see the necessity of accomplishing all recommended visits. Throughout the meetings, we used to inform the community members about the risks pregnant women might face if they did not complete four visits. As we kept disseminating this health message in this village, attitudes kept transforming* (FGD with WG, MDC).

#### Male involvement in maternal and child health

**Fig. 3:**
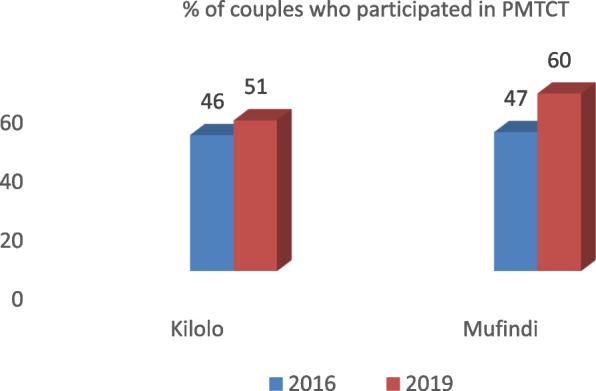
Increased male involvement in maternal and child health

Men's involvement in MCH was said to have had positive consequences for ANC uptake. Specifically, it facilitated increased knowledge of prenatal and postnatal danger signs as well as family planning methods. Ultimately it decreased the barriers related to gender and culture in embracing MCH services. In addition, as part of the strategies for the Prevention of Mother to Child Transmission (PMTCT), couples were expected to participate in HIV/AIDS counselling and testing during the first ANC visit. This is one way of encouraging and promoting male involvement in MCH. Notably, one of the objectives of the IMCHA project was to improve male involvement in PMTCT services. Findings from the facility register revealed that the number of couples participating in PMTCT services increased by 5% and 13% in Kilolo and Mufindi districts, respectively. Figure 3 is illustrative of this.

As captured in Fig. 3, the male involvement increase was not uniform across facilities. For instance, out of the thirteen (13) facilities, ten (10) showed a positive increase, while three (3) facilities recorded a decrease. The facilities with the highest increase were Lyasa in Kilolo District, which recorded a 23% increase, and Iramba in Mufindi District, which recorded a 32% increase. Qualitative findings further revealed changes in attitudes among males as they started accompanying their partners for ANC services. This was articulated by one male champion during FGDs;

*Our presence as male champions during the sensitisation meetings and in other informal gatherings inspired our fellow men to start accompanying their partners to health facilities as something normal. The more we spoke to them about the importance of accompanying their partners, the more their attitudes changed* (IDI with MC MDC).

Similarly, Village Management Teams complimented the views of male champions views as said by one member;

*Much has been evidenced since the implementation of the interventions. For example, the shyness among men to accompany their partners that we used to witness in the past has relatively changed. And the complaints I used to get from healthcare workers over men who don't accompany their partners are also diminishing* (IDI with VMT KDC).

#### Increased postnatal care services

Receiving postnatal care (PNC) within 48 h is important for preventing maternal and infant deaths. This study tracked changes in the utilisation of PNC services. An analysis of quantitative data revealed that at least 95% of women in the intervention villages received PNC within two days after birth. Data showed further that PNC increased by 14% (from 36 to 51%) in Kilolo District and 31% (from 33 to 64%) in Mufindi District. Such changes were also echoed in the IDIs and FGDs conducted with health workers. During interviews, one of the health workers had this to say regarding the increased use of PNC services;

*For the first time at our facility, we started witnessing many clients coming for PNC services. However, further improvement is still needed. Previously, mothers who came were more interested in vaccination for their newborn babies only* (IDI with Healthcare provider in KDC).

#### Women who started using modern family planning methods

**Fig. 4:**
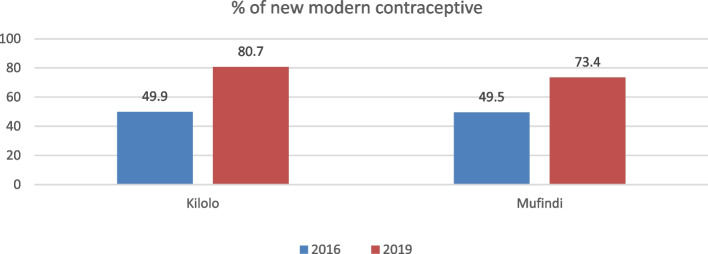
Increase of women using contraceptives

In the public meetings, Women groups identified the challenges related to the women's use of modern family planning methods as one of the key issues affecting the proper uptake of MCH services. It was revealed that women in the intervention villages were not abiding by proper birth spacing, a situation locally referred to as "Kufudikira” in Hehe and Bena languages. As such, WGs strategies focused more on sensitising the community to make use of modern family planning methods. Findings from the study revealed that given the intensive sensitisation approaches, there was a significant improvement in the uptake of family planning methods. Data from the facility registers in Kilolo and Mufindi districts recorded an increase in the number of women who started using family planning methods. This is depicted in Fig. 4.

Findings suggest that the use of modern contraceptives increased by 31% (from 50 to 81%) and 24% (from 49.5% to 73.4%) in Kilolo and Mufindi Districts, respectively. In addition, qualitative findings from IDIs and FGDs attested to an increase in the number of women who used FP. This was attested to the increased number of women who requested health workers to initiate FP for them compared to the previous situation. This was verified by one health worker thus:

*We are now experiencing increasing demand from women wishing to use FP methods more than before. This is also because I have personally participated in sensitisation meetings where we discussed the importance of FP with community members. The good thing is that most of the WGs members have also been role models since those not using FP are now using some methods* (IDI with a health worker, MDC).

### Secondary improvement of maternal and child health services

As the implementation of the interventions gained momentum, other impacts beyond statistical outcomes were revealed. These impacts revolved around the changes experienced at the entire community level, attitude changes among health workers, and empowerment among the implementers of the interventions, as delineated below.

#### Increased awareness and knowledge of matters pertaining to MCH

During interviews and FGDs, participants reaffirmed increased awareness among community members about MCH services, contrary to the situation before the implementation of the interventions. Participants were of the opinion that the number of people who got information through public meetings and home visitation kept increasing. As such, participants articulated different views, which were clear indicators that the awareness of matters pertaining to MCH had increased to a great extent. The implementers of the interventions testified that their understanding of MCH issues increased after their active engagement in implementing the designed strategies. For example, they started to understand the risk associated with the reluctance to follow all prescribed guidelines, which became a starting point for them. In the same vein, the implementers of the intervention explained further that they managed to assist teenage girls who happened to be at school reasonably well. This was facilitated by using the knowledge and understanding they had gained as they participated in implementing the interventions.

Secondly, community members started recognising WGs as an integral part of improving MCH services in their respective jurisdictions. Participants reported that as they continued to implement the interventions, community members got used to calling WG members to visit their households to solve their health problems as well as non-health issues, including gender-based violence. Reinforcing this, one participant attested, *“We were called not once, not twice to different households to solve ANC issues* (FGD with WG member in KDC)". The belief that WGs were able to solve different community issues proves how the community acknowledged their roles. This was followed by the community members' appreciation of the role played by the implementers of the interventions in solving health challenges, especially regarding MCH issues. For example, during FGDs, one of the WG members said, community members desired for the project to have started earlier, they would have benefited a lot more. It was also reported that community members also referred teenage girls who got pregnant to health facilities for ANC services.

The third indicator of increased awareness and knowledge was the increased number of community members who attended sensitisation meetings. It was said that initially, only a few community members participated in the meetings. But as the information about the presence of WGs circulated, the number of attendees in the meetings increased. This was coupled with the increased readiness of community members to inform WGs about pregnant women who had not attended MCH services. This went hand in hand with community members informing WGs about teenage girls who got pregnant and never visited any health facility for ANC services. Overall, participants associated the increased number of community members utilising MCH services with a high level of awareness among the community. Interviews with participants emphasised that instilling awareness in the community simplified the role of health workers who provided MCH services. One health worker succinctly put it:

*I highly appreciate the roles played by the implementers of the interventions. Many community members are now taking steps to come to the facility for ANC services. Through the implemented interventions, every community member knows the dos and don'ts when pregnant* (IDI with health worker in KDC).

#### Change of attitudes among healthcare providers

An analysis of interviews revealed changing patterns of behaviour among health workers who formerly used rude language. Participants disclosed that before the implementation of the interventions, some health workers used to portray a negative attitude towards pregnant women who sought MCH services. First, it was revealed that health workers participated in training, meetings and workshops at different times during the implementation of the interventions. Findings showed that community members started observing changes in the provided services after a series of meetings. This went hand in hand with the strengthening of Health Facility Governing Committees (HFGCs) in all intervention villages. The participation of HFGC in training and sensitisation meetings increased the awareness and understanding of their roles in the whole MCH continuum of care. As such, HFGCs could now closely follow up on all health service provisions, including MCH services.

The second reason for the change of attitude was fueled by health workers' understanding that women group members were aware of the challenges that pregnant women encountered as they sought MCH services. It was revealed that WGs were capable of engaging health workers on these issues, as illustrated by some women during FGDs thus: *“We got a platform to meet with health workers several times and informed them about community perceptions on the provision of MCH services”* (FGD with WG, in MDC). It was revealed further that health workers increased their commitment to the provision of MCH services compared to the situation before, as reinforced by one of the village management team members:

*Healthcare providers have improved the way they handle their clients. As a result, there has been a drastic drop in the number of community members who come to my office complaining about mistreatment. As of now, health workers are aware of the sensitisation meetings conducted monthly in our hamlet to discuss not only issues of the community but also how health workers handle clients* (IDI with VMT, in MDC).

#### Empowerment of women group members

The implementation of WG interventions in 20 villages resulted in the empowerment of women group members. During FGDs with women groups, it was revealed that through participation in the implementation of interventions, they built their capacity to deal with many challenges they could not handle before the interventions. Findings suggested that such empowerment was attained mainly through different activities during the implementation of the interventions. It was revealed that WGs participated in a cycle of four phases that covered eight (8) meetings in the intervention villages. The meetings encompassed WG identifying priority MCH problems as well as prioritising and strategising ways to solve the MCH problems. Through these meetings, WGs got the opportunity to interact and discuss different MCH issues, which broadened their horizons of understanding the causes and impacts of MCH issues and how to address them. For example, during the IDIs with WGS, one participant uttered:

*As supervisors, after being selected, we were trained to communicate with fellow women to convince them to utilise all the required services as pregnant mothers and those who had given birth. The training has been really helpful. Since our recruitment over four years ago, I have managed to closely and cordially interact with health facility workers and cordially with village leaders. I even spoke with the regional medical officer about our work when he visited the community*. (IDI with WGS in KDC).

It was further revealed that WGs also participated in the review meetings and workshops that allowed them to interact and socialise with stakeholders from the village to the regional level. In all these meetings, WGs were allowed to present and discuss their experiences while implementing the interventions. Through these processes, WGs got the opportunity to interact and socialise with community members, family members, district, and regional health managers, and researchers from the University of Dar es Salaam. This, in turn, broadened their skills, self-esteem, courage, confidence and knowledge of WGs, which enabled them to address MCH issues, among others. The analysis of documents, interviews and FGDs revealed different aspects in which WGs experienced empowerment before and after the intervention, as illustrated in Table 4.

**Table 4:** Indicators of WG empowerment

***Kilolo district***	***Mufindi district***
Increased confidence due to high command of respect from community members who enjoyed direct assistance from WGs	Increased understanding of health issues, causes, symptoms and treatment of maternal-related diseases
Started income-generating activities like keeping rabbits, gardening and joining VICOBA	Increased level of women's confidence and independence to do things independently rather than depending entirely on their male partners
Improved the ability to convince their partners to use ANC services	The formation of support groups witnessed improved levels of cooperation among themselves and with community members
Improved the ability to speak before leaders such as regional, district and ward leaders	Increased level of leadership skills. During the election, some women expressed their readiness to compete for leadership positions at the hamlet, village and ward positions
Being the best example in the community (role model). They were dependable in providing counselling services on different issues pertaining to MCH. They were consequently named "*Nurses without uniforms*."	

Some village and ward leaders also confirmed that participation in the women's groups had empowered women. It was noted that women could confidently speak in front of men in meetings. Specifically one participant noted;

*"This research has empowered women in this village. Previously, it took a lot of work for women to speak in meetings. But now, we see women speaking confidently and even asking questions in meetings. This is a great change* (IDI with VMT, KDC).

Another respondent added:

“*Women have now been empowered. I am surprised to see many women now speaking in meetings. We thank the IMCHA team for building confidence of these women*” (IDI with VMT, MDC).

## Discussion

This study examined the effects of community-based interventions in Kilolo and Mufindi districts. The focus was mainly on the changes in intermediate outcomes, such as the uptake of ANC, PNC and FP following the implementation of the IMCHA project. The study findings demonstrate that CBIs through WGs can improve MCH services, as evidenced by an increased percentage of women who started ANC within the first trimester and those who completed four or more ANC visits. Other observed positive changes include the increased number of couples who sought PMTCT services, the increased number of those who received PNC within 48 h as well as the increased uptake of modern FP methods. The effect was also experienced by the implementers of the interventions, who expressed an increased level of awareness and knowledge on matters pertaining to MCH, change of attitudes among health care providers and empowerment of women group members.

The findings are congruent with studies in other LMICs that have demonstrated that CBIs are effective in increasing the uptake of maternal and child health services. For instance, in a systematic review by Sarkar [40] conducted in resource-constrained countries demonstrated how participatory women groups improved ANC check-ups as well as significantly improved of PNC within six weeks. Another WG intervention in Nepal revealed that from baseline to the midline, there was an increase in the women's likelihood of attending ANC at least once during the entire pregnancy period by 7.0 times. The probability of seeking four or more ANC check-ups had doubled [41]. In addition, the participatory women groups in Malawi in SSA reduced neonatal mortality by 33% [28, 42].

The contribution of CBIs may not be proven by only reflecting on statistical measures but also by qualitative changes. As [43] asserted, it is worth noting that the outcomes of CBIs may go beyond quantitative improvement in MCH. As demonstrated in this study, significant behavioural changes were witnessed, including health workers changing attitudes on how to serve their clients, increased community awareness as well as empowerment of WGs. The empowering role of WGs who implement CBIs cannot be underestimated. It is acknowledged that the implementation of WG interventions leads to women’s empowerment and autonomy, thereby leading to the sustainability of the interventions [21–44]. In Nepal, for example, CBIs implemented interventions were said to have increased women's decision-making capacity in the household and improved self-esteem [45, 46]. Likewise, an intervention implemented in South India, referred to as *Kadumbashree,* reported similar findings [43]. Specifically, women were motivated to engage in the implementation of interventions, which was ultimately said to strengthen their confidence and decision-making capacity for their health [47]. Furthermore, a study conducted in Senegal demonstrated significant improvement in women's empowerment as they joined community decision-making groups, easing their decision-making about their healthcare and use of contraception [48]. Members of WGs in Bangladesh are also reported to have gained more knowledge of ANC issues and new skills in managing their savings [40]. Scholars have thus concluded that in LMICs, CBIs that integrate women implementers in interventions are more likely to succeed and yield sustainable outcomes due to the normally applied empowering processes normally [49, 50].

Crafting and designing interventions is critical for the success of the CBIs through women's groups. As revealed in the implemented interventions in Iringa Region, the WGs passed through four phases that enabled them to design and implement several strategies, namely the use of community sensitisation meetings, household visitations of pregnant women, engagement of male champions, engagement of health workers, health facility governing committees, and community gatekeepers Notably, in health services, interventions that focus on holistic improvement of the lives of women yield positive and sustainable result. In addition, evidence has shown that the use of income-generating activities has the potential to increase the retention of members beyond the project grant period and address poverty [51, 52]. This is similar to what was witnessed in the present study where the implementers of the interventions were supported in in income-generating activities and integrating health education with poverty eradication strategies for women. In Nepal, Malawi, Bangladesh and India, for example, women participating in the groups created mother and child health funds, established emergency transport schemes, produced and sold clean delivery kits and initiated vegetable gardens, among other activities [51].

While it is important to attribute the overall improvement of MCH uptake in the intervention villages to the implemented WG interventions in the study area, it is also worth appreciating the government efforts exerted during the implementation of the interventions, specifically from 2015 to 2020. In this period, the government increased investment in the health sector, including the construction of health facilities in Mufindi and Kilolo districts [34, 35]. In addition, during this period, an increased level of civil servants' accountability in the provision of health services was enforced, which led to increased courtesy among health workers as they attended to clients [53, 54]. In addition to the implemented interventions, this might have contributed to the increased uptake of MCH services in the intervention villages and districts. From this perspective, the success of the IMCHA project's success could have received a spillover push from other factors. Nonetheless, on the other hand, the IMCHA project was one of the interventions that contributed to the success of the other aforementioned government interventions due to its timeliness.

## Conclusion

This study aimed at exploring the impacts of CBIs through WGs in rural settings, specifically in Iringa, Tanzania. It has been demonstrated that implementing CBIs through WGs in resource-constrained countries is an important strategy that can help improve MCH services. Impressive results were seen in MCH services in several villages. Furthermore, the study has delineated the impacts beyond the continuum of care of MCH services, including community empowerment and increased knowledge. This is very important if LMIC countries aspire to improve MCH, as espoused in SDGs 3.1 and 3.2. Given the potential benefits of CBIs through WGs, the study recommends that the government of Tanzania and other LMICs should contemplate scaling up the intervention, mostly in rural areas where uptake of MCH is persistently low. It is thus advised further that for a better and smooth implementation of the interventions, there is a need to prepare a manual or guidebook that will provide guidance to regional and district health managers on how to implement WG interventions. It is further recommended that the government ensures that districts and health facilities set aside budgets in their annual health plan to support CBIs through WGs, especially when community members demonstrate their readiness to implement the interventions. However, the effective participation of community members in all phases of the design and implementation of the intervention is of utmost importance.

## Supplementary Information

**Additional file 1.** 

## Data Availability

The datasets generated and analysed during this study are not publicly available since participants did not give consent for public sharing of their information. However, summaries of the information are available from the corresponding author upon reasonable request. The interviews and FGD guides for all study participants are also available upon request.

## Abbreviations

ANC: Antenatal Care
CBIs: Community Based, Interventions
PNC: Postnatal
IMCHA: Innovating for Maternal and Child Health in Africa
PMCT: Prevention of Mother to Child Transmission
WHO: World Health Organisation
WGs: Women Groups
