# Supplementary material for: The effects of community-based interventions on the uptake of selected maternal and child health services: experiences of the IMCHA project in Iringa Tanzania, 2015‐2020
Source: BMC Pregnancy Childbirth. 2023 May 8;23:328. doi: 10.1186/s12884-023-05638-x (PMC10165785; doi:10.1186/s12884-023-05638-x)
Supplement: Supplementary file 1 — Additional file 1. [file 12884_2023_5638_MOESM1_ESM.docx]

**IMCHA PROJECT QUESTIONNAIRE FOR BASELINE DATA ON IMPLEMENTATION OF COMMUNITY-BASED INTERVENTION IN KILOLO AND MUFINDI DISTRICTS.**

**KILOLO DISTRICT BASELINE DATA. January – December 2016**

**Leading Questions**

***A:*** *Biography information of the respondents:*

Age, marital status, education level, occupation.

***B:*** *Status of Maternal and Child Health Service at the facility*

1. Role of as in charge of the Health Facility ……………………
2. How long have you worked here……………………………..
3. When you started working here what was the perspective towards consumption of Antenatal Care Postnatal Care, Family Planning.
4. Do you think the uptake of Antenatal Care Postnatal Care, Family Planning services in your facility is good and why?
5. Are male partners involved in supporting their spouses to utilise the services?
6. How are they involved?
7. What are some of the factors that have been hindering uptake Antenatal Care Postnatal Care, Family Planning services in your facility is good and why?
8. What are some of the suggestions that you feel might improve uptake of services?
9. With aid of facility register, please help identify the indicators of the following MCH service in your facility: (i) antenatal care, Postnatal Care, Male involvement and Family Planning

| **INDICATORS** | **NAME OF FACILITY** | | | | | **TOTAL** |
| --- | --- | --- | --- | --- | --- | --- |
|  | **IBUMU** | **ITUNGI** | **MAZOMBE** | **UKUMBI** | **LYASA** |  |
| **Antenatal Care** |  |  |  |  |  |  |
| Started ANC within the first trimester (12 weeks) |  |  |  |  |  |  |
| Started ANC after 12 weeks of pregnancy |  |  |  |  |  |  |
| Number of women attended first ANC |  |  |  |  |  |  |
| Completed 4 or more visits |  |  |  |  |  |  |
| **Male Involvement** |  |  |  |  |  |  |
| Number of women participated in PMTCT |  |  |  |  |  |  |
| Number of couple participated in PMTCT |  |  |  |  |  |  |
| Number of couple attended first ANC |  |  |  |  |  |  |
| **Postnatal Care** |  |  |  |  |  |  |
| Women who received PNC within 2 days |  |  |  |  |  |  |
| Women who received PNC within 3-7 days |  |  |  |  |  |  |
| **Family Planning** |  |  |  |  |  |  |
| Number of Women started using short term method of Family Planning (10 – 24yrs) |  |  |  |  |  |  |
| Number of Women started using short term method of Family Planning (24yrs and above) |  |  |  |  |  |  |
| Number of Women started using Long term/permanent method of Family Planning (10 – 24yrs) |  |  |  |  |  |  |
| Number of Women started using modern method of contraception (short and Long term methods) (10 – 24yrs) |  |  |  |  |  |  |
| Number of Women started using modern method of contraception (short and Long term methods) (24yrs and above) |  |  |  |  |  |  |

**IMCHA PROJECT QUESTIONNAIRE FOR BASELINE DATA ON IMPLEMENTATION OF COMMUNITY-BASED INTERVENTION IN KILOLO AND MUFINDI DISTRICT.**

**KILOLO DISTRICT ENDLINE LINE DATA June 2018 – May 2019**

***A:*** *Biography information of the respondents:*

Age, marital status, education level, occupation.

***B:*** *Status of Maternal and Child Health Service at the facility*

1. Role of the Health facility In-charge ……………………..
2. How long have you worked here…………………………..
3. For the past three years, what has been the level of utilisation of Maternal and Child health Services?
4. Do you think the uptake of Antenatal Care Postnatal Care, Family Planning services in your facility has improved in the past three years? And why?
5. If yes, do you think role of none state actors like the implemented IMCHA project has played the role of its improvement?
6. Are male partners involved in supporting their spouses to utilise the services?
7. What are some of the factors that have been hindering uptake Antenatal Care Postnatal Care, Family Planning services in your facility is good and why?
8. With aid of facility register, please help identify the indicators of the following MCH service in your facility: (i) antenatal care, Postnatal Care, Male involvement and Family Planning

| **INDICATORS** | **NAME OF FACILITY** | | | | | **TOTAL** |
| --- | --- | --- | --- | --- | --- | --- |
|  | **IBUMU** | **ITUNGI** | **MAZOMBE** | **UKUMBI** | **LYASA** |  |
| **Antenatal Care** |  |  |  |  |  |  |
| Started ANC within the first trimester (12 weeks) |  |  |  |  |  |  |
| Started ANC after 12 weeks of pregnancy |  |  |  |  |  |  |
| Number of women attended first ANC |  |  |  |  |  |  |
| Completed 4 or more visits |  |  |  |  |  |  |
| **Male Involvement** |  |  |  |  |  |  |
| Number of women participated in PMTCT |  |  |  |  |  |  |
| Number of couple participated in PMTCT |  |  |  |  |  |  |
| Number of couple attended first ANC |  |  |  |  |  |  |
| **Postnatal Care** |  |  |  |  |  |  |
| Women who received PNC within 2 days |  |  |  |  |  |  |
| Women who received PNC within 3-7 days |  |  |  |  |  |  |
| **Family Planning** |  |  |  |  |  |  |
| Number of Women started using short term method of Family Planning (10 – 24yrs) |  |  |  |  |  |  |
| Number of Women started using short term method of Family Planning (24yrs and above) |  |  |  |  |  |  |
| Number of Women started using Long term/permanent method of Family Planning (10 – 24yrs) |  |  |  |  |  |  |
| Number of Women started using modern method of contraception (short and Long term methods) (10 – 24yrs) |  |  |  |  |  |  |
| Number of Women started using modern method of contraception (short and Long term methods) (24yrs and above) |  |  |  |  |  |  |

**IMCHA PROJECT QUESTIONNAIRE FOR BASELINE DATA ON IMPLEMENTATION OF COMMUNITY-BASED INTERVENTION IN KILOLO AND MUFINDI DISTRICT**

**MUFINDI DISTICT BASELINE DATA**

**Leading Questions**

***A:*** *Biography information of the respondents:*

Age, marital status, education level, occupation.

***B:*** *Status of Maternal and Child Health Service at the facility*

1. Role of Official……………………………………………………..
2. How long have you worked here…………………………..
3. When you started working here what was the perspective towards consumption of Antenatal Care Postnatal Care, Family Planning
4. Do you think the uptake of Antenatal Care Postnatal Care, Family Planning services in your facility is good and why?
5. Are male partners involved in supporting their spouses to utilise the services?
6. How are they involved?
7. What are some of the factors that have been hindering uptake Antenatal Care Postnatal Care, Family Planning services in your facility is good and why?
8. What are some of the suggestions that you feel might improve uptake of services

| **INDICATORS** | **NAME OF FACILITY** | | | | | **TOTAL** |
| --- | --- | --- | --- | --- | --- | --- |
|  | **IKIMILINZOWO** | **IRAMBA** | **KASANGA** | **KIBENGU** | **NYOLOLO** |  |
| **Antenatal Care** |  |  |  |  |  |  |
| Started ANC within the first trimester (12 weeks) |  |  |  |  |  |  |
| Started ANC after 12 weeks of pregnancy |  |  |  |  |  |  |
| Number of women attended first ANC |  |  |  |  |  |  |
| Completed 4 or more visits |  |  |  |  |  |  |
| **Male Involvement** |  |  |  |  |  |  |
| Number of women participated in PMTCT |  |  |  |  |  |  |
| Number of couple participated in PMTCT |  |  |  |  |  |  |
| Number of couple attended first ANC |  |  |  |  |  |  |
| **Postnatal Care** |  |  |  |  |  |  |
| Women who received PNC within 2 days |  |  |  |  |  |  |
| Women who received PNC within 3-7 days |  |  |  |  |  |  |
| **Family Planning** |  |  |  |  |  |  |
| Number of Women started using short term method of Family Planning (10 – 24yrs) |  |  |  |  |  |  |
| Number of Women started using short term method of Family Planning (24yrs and above) |  |  |  |  |  |  |
| Number of Women started using Long term/permanent method of Family Planning (10 – 24yrs) |  |  |  |  |  |  |
| Number of Women started using modern method of contraception (short and Long term methods) (10 – 24yrs) |  |  |  |  |  |  |
| Number of Women started using modern method of contraception (short and Long term methods) (24yrs and above) |  |  |  |  |  |  |

**IMCHA PROJECT QUESTIONNAIRE FOR BASELINE DATA ON IMPLEMENTATION OF COMMUNITY-BASED INTERVENTION IN KILOLO AND MUFINDI DISTRICT**

**MUFINDI DISTICT ENDLINE DATA June 2018 – May 2019**

***A:*** *Biography information of the respondents:*

Age, marital status, education level, occupation.

***B:*** *Status of Maternal and Child Health Service at the facility*

1. Role of the Health facility In-charge ……………………..
2. How long have you worked here…………………………..
3. For the past three years, what has been the level of utilisation of Maternal and Child health Services?
4. Do you think the uptake of Antenatal Care Postnatal Care, Family Planning services in your facility has improved in the past three years? And why?
5. If yes, do you think role of none state actors like the implemented IMCHA project has played the role of its improvement?
6. Are male partners involved in supporting their spouses to utilise the services?
7. What are some of the factors that have been hindering uptake Antenatal Care Postnatal Care, Family Planning services in your facility is good and why?
8. With aid of facility register, please help identify the indicators of the following MCH service in your facility: (i) antenatal care, Postnatal Care, Male involvement and Family Planning

| **INDICATORS** | **NAME OF FACILITY** | | | | | **TOTAL** |
| --- | --- | --- | --- | --- | --- | --- |
|  | **IKIMILINZOWO** | **IRAMBA** | **KASANGA** | **KIBENGU** | **NYOLOLO** |  |
| **Antenatal Care** |  |  |  |  |  |  |
| Started ANC within the first trimester (12 weeks) |  |  |  |  |  |  |
| Started ANC after 12 weeks of pregnancy |  |  |  |  |  |  |
| Number of women attended first ANC |  |  |  |  |  |  |
| Completed 4 or more visits |  |  |  |  |  |  |
| **Male Involvement** |  |  |  |  |  |  |
| Number of women participated in PMTCT |  |  |  |  |  |  |
| Number of couple participated in PMTCT |  |  |  |  |  |  |
| Number of couple attended first ANC |  |  |  |  |  |  |
| **Postnatal Care** |  |  |  |  |  |  |
| Women who received PNC within 2 days |  |  |  |  |  |  |
| Women who received PNC within 3-7 days |  |  |  |  |  |  |
| **Family Planning** |  |  |  |  |  |  |
| Number of Women started using short term method of Family Planning (10 – 24yrs) |  |  |  |  |  |  |
| Number of Women started using short term method of Family Planning (24yrs and above) |  |  |  |  |  |  |
| Number of Women started using Long term/permanent method of Family Planning (10 – 24yrs) |  |  |  |  |  |  |
| Number of Women started using modern method of contraception (short and Long term methods) (10 – 24yrs) |  |  |  |  |  |  |
| Number of Women started using modern method of contraception (short and Long term methods) (24yrs and above) |  |  |  |  |  |  |
